# Postsynaptic protein synthesis is required for presynaptic enhancement in persistent forms of long-term potentiation

**DOI:** 10.3389/fnsyn.2013.00001

**Published:** 2013-02-27

**Authors:** Victoria P. A. Johnstone, Clarke R. Raymond

**Affiliations:** Department of Neuroscience, The John Curtin School of Medical Research and Eccles Institute of Neuroscience, The Australian National UniversityCanberra ACT, Australia

**Keywords:** LTP, expression, presynaptic, hippocampus, electrophysiology

## Abstract

Long-term potentiation (LTP) in the hippocampus is a fundamental process underlying learning and memory in the brain. At CA3-CA1 synapses, three discrete forms of LTP (LTP1, 2, and 3) have been differentiated on the basis of their persistence, maintenance mechanisms, Ca^2+^ signaling pathways, expression loci, and electrophysiological requirements. We previously showed that LTP2 and LTP3 involve a presynaptic expression component that is established in a translation-dependent manner. Here we investigate the locus of translation required for presynaptic expression. Neurotransmitter release rate was estimated via FM 1-43 destaining from CA3 terminals in hippocampal slices from male Wistar rats (6–8 weeks). Destaining was measured at sites making putative contact with CA1 dendritic processes in stratum radiatum that had been filled with a membrane impermeable translation inhibitor and a fluorescent indicator. Our results suggest that inhibition of postsynaptic translation eliminates the enhanced release ordinarily observed at 160 min post-LTP induction, and that this effect is limited to sites closely apposed to the filled postsynaptic cell. We conclude that postsynaptic translation is required for the presynaptic component of LTP2 and LTP3 expression. These data considerably strengthen the mechanistic separation of LTP1, 2, and 3 and provide evidence for an expanded repertoire of communication between synaptic elements.

## Introduction

Long-term potentiation (LTP) is an activity-dependent and persistent increase in synaptic strength and is a model paradigm in the investigation of hippocampal learning and memory (Bliss et al., 2003). Although extensively studied, the existence of multiple LTP types even at a single class of synapses has constrained progress in understanding LTP function. We have recently expanded upon a model in which synapses between CA3 and CA1 pyramidal neurons support at least three mechanistically discrete forms of LTP, termed LTP1, 2, and 3 (Abraham and Otani, 1991; Raymond, 2007; Reymann and Frey, 2007). These forms of LTP are discernible from classical E-LTP and L-LTP (Frey et al., 1993, 1988) in that they can apparently be selectively and independently induced via Ca^2+^ signals derived from unique sources and intracellular compartments. In addition, LTP1, 2, and 3 differ in their persistence, their electrophysiological requirements for induction, the mechanisms and locus of expression, and their dependence on new protein synthesis for long-term maintenance (Raymond and Redman, 2002, 2006; Raymond, 2008; Johnstone and Raymond, 2011). Briefly, LTP1 is a short-lived form of LTP that requires RyR-mediated Ca^2+^ release in the postsynaptic spine compartment for its induction, is expressed postsynaptically but not presynaptically, and is independent of protein synthesis and gene transcription. In contrast LTP2 is more persistent, requires Ca^2+^ via dendritic IP_3_Rs for its induction, is dependent on translation but not transcription for its persistence, and recruits a presynaptic component to its expression repertoire by 160 min post-induction. Finally LTP3 is a very persistent form of LTP that requires somatic L-VGCC activation for its induction, is dependent on both translation and transcription for its maintenance, and involves a presynaptic enhancement of neurotransmitter release that is detectable within 80 min post-induction.

We have recently established that the presynaptic enhancement associated with LTP2 and LTP3 involves NO signaling and transcription-independent protein synthesis (Johnstone and Raymond, 2011). Therefore, the translation required for the presynaptic component of both forms of LTP could occur either postsynaptically, (upstream, downstream or in parallel with NO signaling), or presynaptically in response to NO. Local postsynaptic protein synthesis has been extensively characterized (Sutton and Schuman, 2006) and is often associated with LTP. There is also mounting evidence to support the occurrence of translation in presynaptic terminals (reviewed by Akins et al., 2009). Local presynaptic protein synthesis has been established in both immature and mature rat hippocampal cultures (Sebeo et al., 2009), in forms of LTP and long-term depression in *Xenopus* nerve-muscle cultures (Zhang and Poo, 2002), in corticostriatal fibers (Yin et al., 2006) and in hippocampal mossy fiber-CA3 synapses (Huang and Hsu, 2004). Here we present results demonstrating that the enhanced exocytosis associated with LTP2 and 3 is dependent on protein synthesis within the postsynaptic cell.

## Materials and methods

### Ethical approval

Experiments were performed in accordance with the Australian National University Animal Experimentation Ethics Committee guidelines.

### Slice preparation and electrophysiology

Male Wistar rats (6–8 weeks) were anaesthetized with isofluorane, decapitated, and the brains submerged in ice-cold dissecting solution (mM: 124 NaCl, 3.2 KCl, 1.25 NaH_2_PO_4_, 26 NaHCO_3_, 0.5 CaCl_2_, 7 MgCl_2_, 2 ascorbate, 3 pyruvate and 10 D-glucose, equilibrated with 95% O_2_ – 5% CO_2_). Transverse hippocampal slices (300 μm) were prepared using a vibratome and area CA3 was removed to reduce potential hyperexcitability. Slices were transferred to a holding solution as above, except the Ca^2+^, Mg^2+^, and d-glucose concentrations were adjusted to 2.5 mM, 1.3 mM, and 25 mM respectively. Slices were maintained at 34°C for at least 30–40 min, then at room temperature for a further 30 min, or until required.

Slices were perfused in a continuous flow (~2 ml min^−1^) of recording solution (as per holding solution, but without ascorbate and pyruvate) at 32–34°C. Whole-cell voltage-clamp recordings from CA1 pyramidal cells were made using glass electrodes (4–6 MΩ) filled with (mM): 127 KMeSO_4_, 8 KCl, 10 Hepes, 10 sodium phosphocreatine, 4 Mg-ATP, 0.4 Na-GTP, 0.05 Alexa Fluor 488 and in some cases 0.0035 gelonin (Enzo Life Sciences). Synaptic responses were evoked by stimulation of the Schaffer collateral/commissural axons (0.1 ms-pulse-width) with a Teflon-insulated tungsten bipolar electrode placed 100–200 μm from the cell of interest. The stimulation amplitude was adjusted in current-clamp to produce EPSPs approximately 1/3rd of action potential threshold. EPSC recordings (200–300 pA) were then obtained with somatic membrane potential clamped at −65 mV.

**Figure 1:**
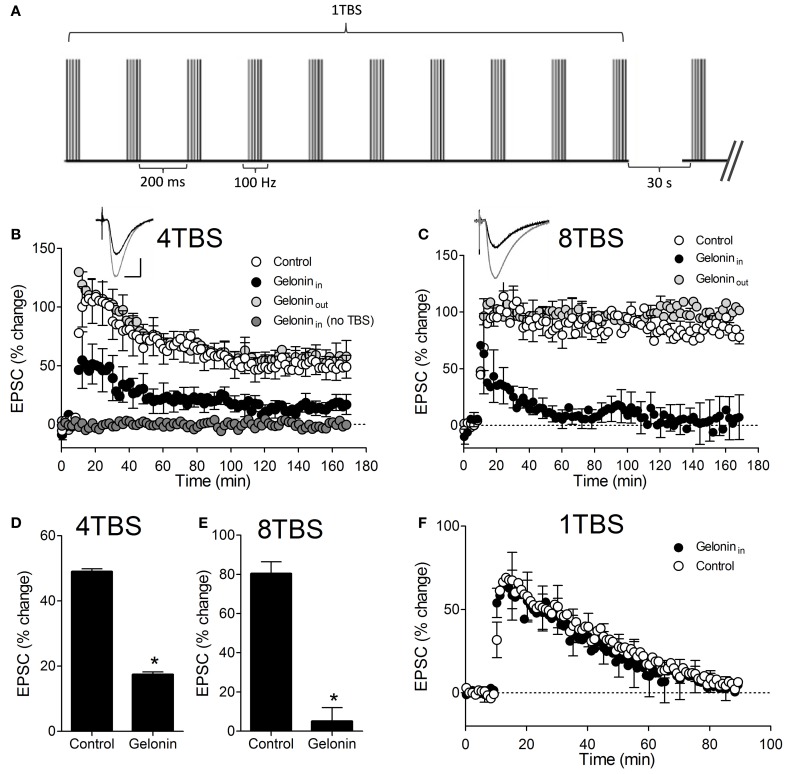
**LTP2 and LTP3 persistence and magnitude is reduced with gelonin in the patch pipette. (A)** Pictorial representation of the TBS protocol used to induce LTP1, 2, and 3. When more than one train was used (i.e., 4TBS and 8TBS) there was a 30 s inter-train interval. All TBS trains were delivered at the test-pulse intensity. **(B)** Mean percent change in EPSC amplitude following 4TBS and 8TBS **(C)** in control cells and cells filled with the membrane impermeable translation inhibitor gelonin (gelonin_in_). Inset shows example EPSC traces before (black) and immediately after (gray) delivery of 4TBS or 8TBS in the control situation. Scale bar indicates 200 μA and 10 ms. Inclusion of gelonin in the patch pipette significantly reduced LTP magnitude at 160 min post-4TBS **(D)** and post-8TBS **(E)** (^*^p < 0.05). Gelonin did not affect EPSC recordings in the absence of TBS **(A)** nor did it affect weak LTP1 induced by 1TBS **(F)** or LTP2 and LTP3 when applied extracellularly (gelonin_out_) **(B** and **C)**.

Baseline recordings (10 min) were initiated no later than 5 min after whole-cell configuration was obtained. Baseline synaptic responses were evoked by delivery of stimuli at a frequency of 0.066 Hz. Every 2.5 min a second pulse was administered 20 ms after the first to enable measurements of paired-pulse facilitation (PPF) and the paired-pulse ratio (PPR). Series resistance was monitored on-line and recordings were terminated if this varied by more than 20% from the baseline value, or alternatively rose above 25 MΩ. LTP was induced in current-clamp by theta-burst stimulation (TBS), consisting of trains of 10 × 100 Hz bursts (5 pulses/burst) with a 200 ms interburst interval (at the test pulse intensity). One train of ten bursts is denoted 1TBS. When multiple trains were delivered (i.e., 4TBS and 8TBS) they were separated by 30 s. See Figure 1A for pictorial representation of the induction stimulus. Synaptic responses were monitored for at least 160 min post-LTP induction, with a success rate of maintaining stable recordings over this period of approximately 15%.

Unless otherwise stated, two-tailed Student's *t*-tests were used to determine statistical significance at the 95% confidence level.

### FM 1-43 loading and unloading

After recording stable Schaffer collateral-evoked EPSCs for 160 min post-TBS, synaptic vesicles were loaded by delivering a train of 1200 stimuli (at 10 Hz) in the presence of FM 1-43 (5 μM, Biotium Inc.) and D-APV (100 μM, Tocris; Figure 1B). The dye application continued for 1 min beyond the duration of the loading stimulus to ensure completion of endocytosis and dye uptake. FM 1-43 was then washed out in the presence of ADVASEP-7 (0.5 mM, Biotium Inc.) for 20 min to remove any extracellularly bound dye (Kay et al., 1999). Terminals making putative contact with spines along an Alexa Fluor 488-labeled dendritic shaft (100–200 μm from the soma) were identified and destaining was achieved by stimulating at 1 Hz for 6 min in the presence of ADVASEP-7 and D-APV. Results from preliminary experiments demonstrated that this protocol maximally unloaded the dye at a rate that was dependent on Ca^2+^, temperature and stimulation frequency, consistent with it being a valid measure of exocytosis (data not shown).

### Two-photon imaging

Fluorescence imaging (of labeled release sites and filled dendritic processes) was performed using a Zeiss LSM 510 two-photon laser-scanning microscope with a water immersion objective (Achroplan 40×/0.75 N.A.). Two-photon excitation was achieved with a Ti:Sapphire laser tuned to 810 nm. Imaging at greater depths than is normally ideal for monitoring FM 1-43 release was necessary in these experiments in order to track dendrites at distances of approximately 150–250 μm from the soma. All fields imaged were typically 70–100 μm deep, and were 40–80 μm away from the stimulating electrode. A series of 5–10 images (512 × 512, 0.15 μm/pixel in the *x*-*y* axes) were taken at 0.2–0.4 μm intervals in the *z*-plane. These *z*-stacks were repeated every 30 s, beginning 1 min prior to and during the unloading stimulation. In offline analyses, maximal *z*-projections were generated from each image series to create one image per 30 s time point. Circular regions of interest were defined around the center of brightly stained punctate fluorescent spots and 2–16 terminals and 3 background regions were measured at each time point. A smaller number of puncta were sampled here than is usually possible with FM 1-43 loading as a result of the depth at which imaging was performed. The distance from each punctum to the nearest spine/dendritic shaft was also measured. Only puncta that satisfied four predetermined criteria were included in analysis. These were: (1) diameter of 0.5–1 μm; (2) approximately circular shape; (3) minimal *x-y* movement (such that any puncta that moved beyond the specified region of interest was excluded); and (4) activity-dependent destaining (i.e., no spontaneous destaining) that was well fit by a first order exponential decay function. A fluorescence time-course was generated by subtracting the average background fluorescence at each time point then normalizing the fluorescence of each punctum at each time point to the average of the prestimulus values. Photobleaching was corrected for by normalizing to the average background fluorescence at the corresponding time point throughout the unload stimulation. The halftime of decay of fluorescence intensity during unloading (*t*_1/2_) was calculated for each punctum from single exponential decay curves fitted to the 6 min of stimulus-induced destaining.

## Results

### LTP2 and LTP3 are dependent on postsynaptic translation for their persistence

In order to establish the locus of translation that is required for the persistence of LTP2 and LTP3, individual CA1 neurons were filled with a membrane impermeable translation inhibitor (gelonin, 3.5 μM). Gelonin inhibits protein synthesis by inactivating the 60S ribosome (Stirpe et al., 1980) and has previously been used to inhibit local postsynaptic protein synthesis required for learning-related facilitation (Sherff and Carew, 2004; Villareal et al., 2007) as well as local presynaptic translation required for synaptic plasticity in nerve-muscle cultures (Zhang and Poo, 2002).

LTP2 was significantly inhibited by the inclusion of gelonin in the whole-cell patch pipette (Figure 1B). Since LTP is usefully classified on the basis of persistence, the post-TBS data were fit with a double exponential decay function as described previously (Raymond et al., 2000; Raymond and Redman, 2002). The mean time-constant of decay of the slower exponential (τ) was used as a measure of LTP persistence and any instances in which data were not well fit were excluded from any further analysis. LTP2 τ was significantly reduced from 99 ± 4 min (*n* = 6) to 34 ± 2 min (*n* = 9; *p* < 0.05) with gelonin in the patch pipette. The amplitude of the EPSC in the last 5 min of recording was also significantly reduced with the inclusion of gelonin, from 49 ± 1% (*n* = 6) to 17 ± 1% (*n* = 9; Figure 1D; *p* < 0.05). This is consistent with previously reported data demonstrating a role for translation in the persistence of LTP2 (Raymond et al., 2000; Johnstone and Raymond, 2011). Similarly, both LTP3 persistence and final magnitude were reduced by inhibition of translation with gelonin (Figure 1C). Interestingly, the initial magnitude of both LTP2 and LTP3 was decreased with gelonin in the patch pipette; a result that may provide insight into the critical role of early translation immediately post-LTP. A τ value for 8TBS LTP could not be determined in these experiments because all control LTP was essentially non-decremental over the recording period and could not be fit by exponential decay curves. We therefore restricted our analysis to comparison of LTP magnitude over the last 5 min of recording. Gelonin significantly reduced LTP3 magnitude at 160 min post-8TBS (Figure 1E; Control = 80 ± 6%, *n* = 4; gelonin = 5 ± 7%, *n* = 5; *p* < 0.05). Although inhibition of translation with gelonin also reduced the LTP2 and LTP3 EPSC amplitude immediately post-induction, we conclude that this is unlikely to be due to local toxicity effects of the drug. In the absence of TBS, EPSC amplitudes were unaffected by gelonin in the patch pipette over the entire recording period (*n* = 4; Figure 1B) and gelonin had no effect on the induction of translation-independent LTP1 (*n* = 4; Figure 1F). Importantly, application of gelonin extracellularly (100 μM) did not affect the persistence of LTP2 and LTP3 (*n* = 3, Figures 1B,C), confirming gelonin as a membrane-impermeable compound and supporting the postsynaptic specificity of effects described above. These results suggest that inhibition of protein synthesis in the postsynaptic compartment is sufficient to curtail the expression and maintenance of LTP2 and LTP3.

### Presynaptic LTP expression requires postsynaptic translation

**Figure 2:**
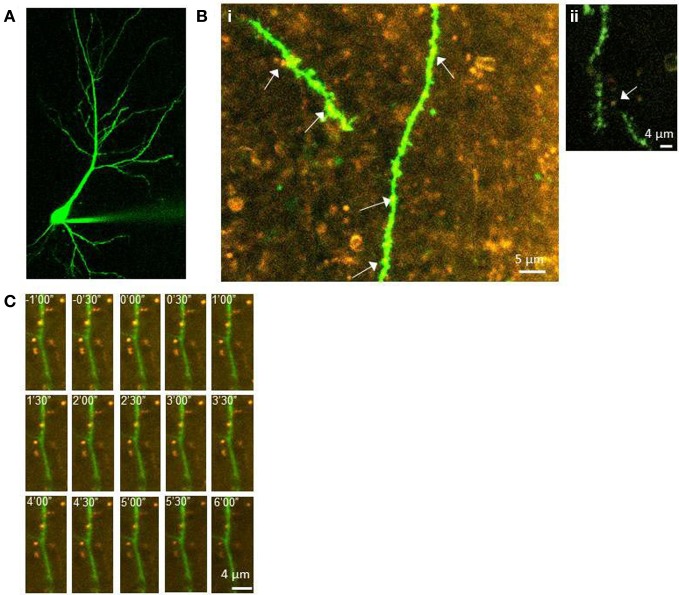
**Labeling of pre- and postsynaptic elements of CA3-CA1 synapses. (A)** CA1 neuron filled with Alexa Fluor 488. **(B)** Examples of FM 1-43 loaded terminals (orange) in the same field of view as Alexa Fluor 488 filled CA1 dendritic branches (green). Arrows indicate putative contacts between terminals and spines/dendrites. The dendritic branch is approximately 140 μm from the soma in **i**, and 180 μm from the soma in **ii**. **(C)** Example time-series of destaining during the unload period in an experiment in which gelonin was included. Images shows sequential loss of FM 1-43 from stained terminals. Numbers indicate time in minutes and seconds, with zero representing the onset of unloading stimulation (1 Hz, 6 min).

**Figure 3:**
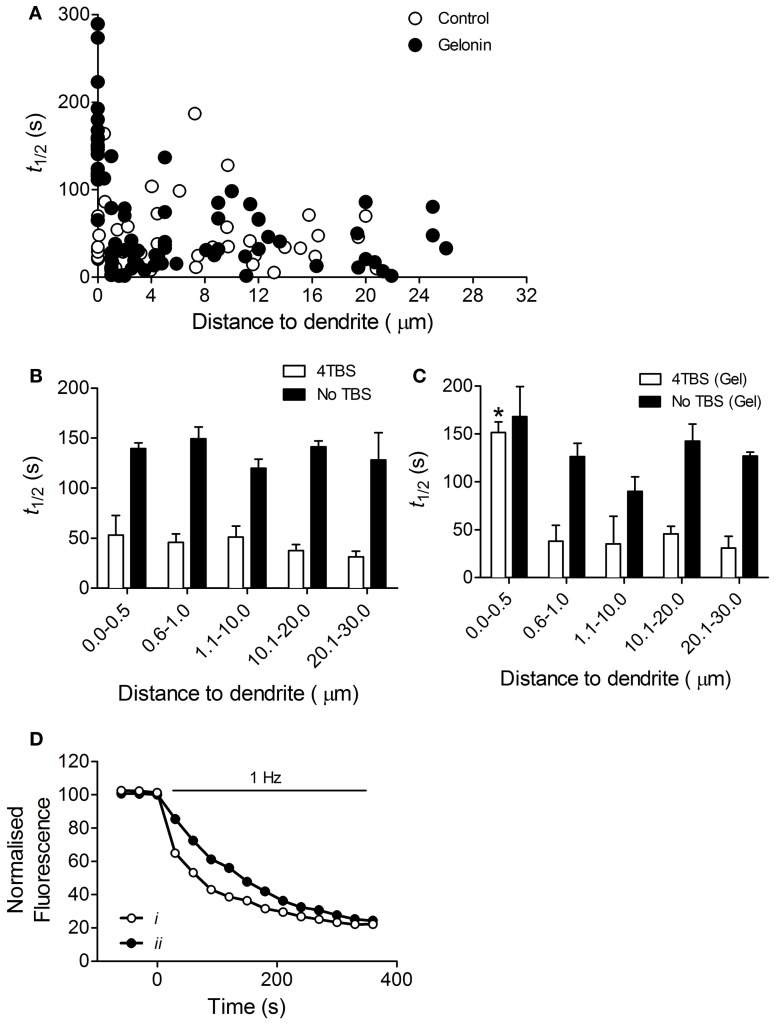
**Enhanced presynaptic function associated with LTP2 is dependent on postsynaptic translation. (A)** Scatter plot showing the half-life of FM 1-43 destaining versus distance to filled dendrite for individual terminals in slices stimulated with 4TBS with and without gelonin in the patch pipette. **(B)** Histogram summarizing destaining rate data from the “Control” group in **(A)** and from “No TBS” slices with distances binned. Destaining rate was enhanced in slices stimulated with 4TBS and there was no effect of distance to nearest postsynaptic dendrite in these untreated cells. **(C)** Histogram of data obtained from 4TBS gelonin-treated group in **(A)** and “No TBS” slices treated with gelonin, with distances binned. Inclusion of gelonin in the patch pipette abolished enhanced release induced by 4TBS only at sites close to (0–0.5 μm) the filled postsynaptic cell, but had no effect on release in the absence of TBS (One-Way ANOVA, ^*^p < 0.05). **(D)** Examples of destaining from two representative puncta in the 0.0–0.5 μm distance group. ***i***, control terminal with no gelonin in the pipette shows normal, LTP-related enhanced rate of dye release. ***ii***, terminal with gelonin in the postsynaptic cell shows reduced rate of dye release.

To determine whether postsynaptic translation is also required for the enhanced presynaptic release associated with LTP2 and LTP3, FM 1-43 destaining was measured from CA3 terminals at various distances from CA1 apical dendrites filled with Alexa Fluor 488 and gelonin. This technique allowed for comparisons of presynaptic function between distant terminals and those in putative contact with the gelonin-treated cell. Figure 3 shows an example of a CA1 neuron filled with Alexa Fluor 488 (Figure 2A) and of FM 1-43 filled CA3 terminals surrounding filled postsynaptic dendritic branches (Figure 2B). Our previous data suggests that our sampled puncta were representative of CA3 terminals, and did not include large numbers of GABAergic inhibitory interneurons or astrocytes (Johnstone and Raymond, 2011). An example time series of FM 1-43 release under our conditions can be seen in Figure 2C.

**Figure 4:**
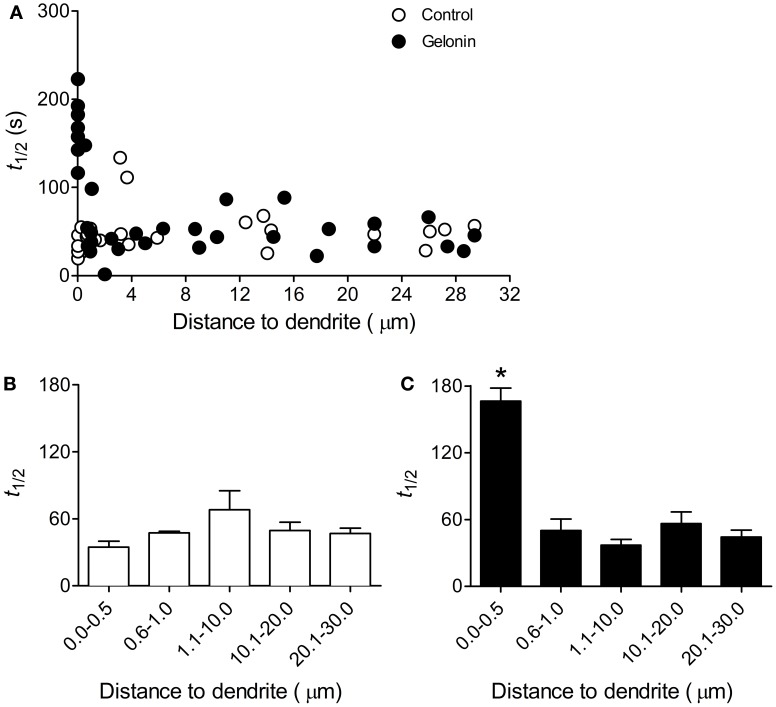
**Enhanced presynaptic function associated with LTP3 is dependent on postsynaptic translation. (A)** Scatter plot showing the half-life of FM 1-43 destaining versus distance to filled dendrite for individual terminals in slices stimulated with 8TBS with and without gelonin in the patch pipette. **(B)** Histogram summarizing destaining rate data obtained from the “Control” group in **(A)** except with distances binned. There was no effect of distance on mean half-life of destaining in untreated cells. **(C)** Histogram summarizing data obtained from “gelonin” group in **(A)** except with distances binned. Inclusion of gelonin in the patch pipette abolished enhanced release induced by 8TBS only at sites close to the treated postsynaptic cell (One-Way ANOVA, ^*^p < 0.05).

In control slices an increased rate of exocytosis was measured at all terminals following induction of LTP2 with 4TBS, regardless of distance to the dendrite (*n* = 40 terminals, six slices; Figures 3A,B). With gelonin in the postsynaptic recording pipette enhanced release was abolished only at sites very close to the postsynaptic cell (0–0.5 μm; *n* = 84 terminals, nine slices; Figures 3A,C; One-Way ANOVA, *F*_(4, 77)_ = 35.02; *p* < 0.05). Enhanced exocytosis remained intact at sites further removed from the treated postsynaptic cell. Example data depicting the time course of destaining from individual puncta with and without gelonin are shown in Figure 3D. The enhanced exocytosis associated with LTP3 was also found to be dependent on postsynaptic translation. In control slices enhanced release was observed following 8TBS regardless of distance to the nearest filled dendrite (*n* = 39 terminals; four slices; Figures 4A,B). However, inhibition of postsynaptic translation with gelonin (*n* = 34 terminals, five slices) blocked enhanced release at 160 min post-8TBS only at distances between 0 and 0.5 μm to the nearest filled dendrite (Figures 4A,C; One-Way ANOVA, *F*_(4, 29)_ = 37.06; *p* < 0.05). As was the case with LTP2, enhanced release remained unaffected at sites further removed from the treated postsynaptic cell. Importantly, destaining rate in the absence of TBS remained unchanged both with (*n* = 39 terminals, four slices) and without gelonin (*n* = 17 terminals, three slices), regardless of distance to the nearest filled CA1 dendrite (Figures 3B,C).

### Paired-pulse facilitation data support a role for presynaptic expression that is dependent on postsynaptic translation

As a complementary experiment, levels of PPF were recorded with and without gelonin before and at various time points following induction of LTP2 and 3. A reduction in the PPR is thought to be indicative of increased Pr (Schulz et al., 1994, 1995; Sokolov et al., 1998, 2002), therefore this analysis was valuable as it allowed for comparison of release probability with FM 1-43 destaining at 80 min and 160 min post-TBS.

**Figure 5:**
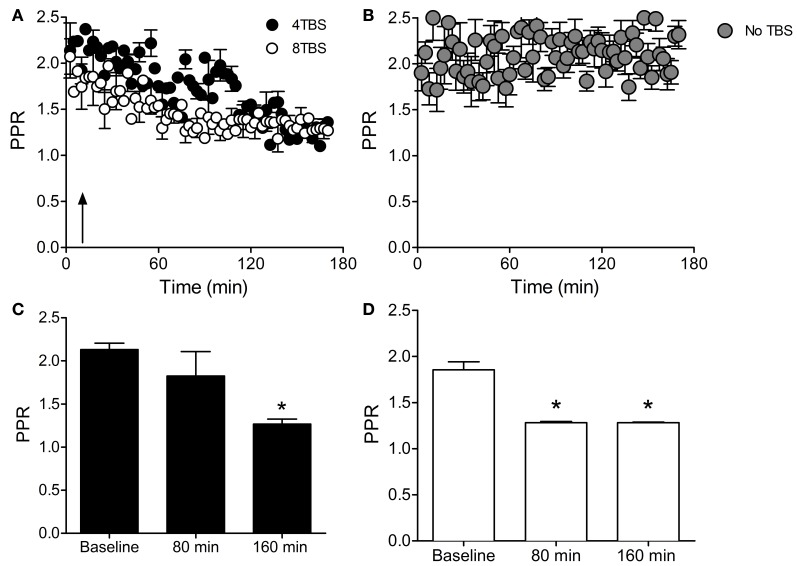
**PPR measurements demonstrate increased release probability with LTP2 and LTP3. (A)** Paired-pulse ratio (PPR) in slices stimulated with 4TBS and 8TBS (at time point indicated by arrow) showing a reduction in the ratio over time for both groups. **(B)** PPR was stable in the absence of TBS. **(C)** Bar graph showing mean PPR during the first 10 min of recording (Baseline) and at 80 min and 160 min post-4TBS. PPR was significantly reduced at 160 min post-4TBVS, consistent with the enhanced FM 1-43 destaining data for LTP2. **(D)** Bar graph as for **(C)** except for LTP3 data induced with 8TBS. PPR was significantly reduced at both 80 min and 160 min post-TBS, consistent with the FM 1-43 destaining data for LTP3. ^*^Indicates 0.05

LTP2 was associated with a reduction in PPR at 160 min after induction (PPR Baseline = 2.13 ± 0.07, *n* = 5 cells; PPR 160 min = 1.26 ± 0.05, *n* = 5 cells; *p* < 0.05), but not at 80 min (PPR 80 min = 1.82 ± 0.28, *n* = 5 cells; Figures 5A,C). LTP3 was associated with a reduction in PPR at both 80 min (PPR Baseline = 1.85 ± 0.08, *n* = 4 cells; PPR 80 min = 1.28 ± 0.01, *n* = 4 cells) and 160 min post-TBS (PPR 160 min = 1.28 ± 0.005, *n* = 4 cells; Figures 5A,D; *p* < 0.05). Importantly, the PPR remained stable over the entire recoding period in the absence of TBS (Figure 5B). These data are consistent with our previously reported FM 1-43 destaining data, and support the finding that LTP2 involves a presynaptic component that is not expressed until 80–160 min post-TBS, whereas LTP3 involves a presynaptic component that may be recruited earlier and is apparent as early as 80 min post-TBS.

**Figure 6:**
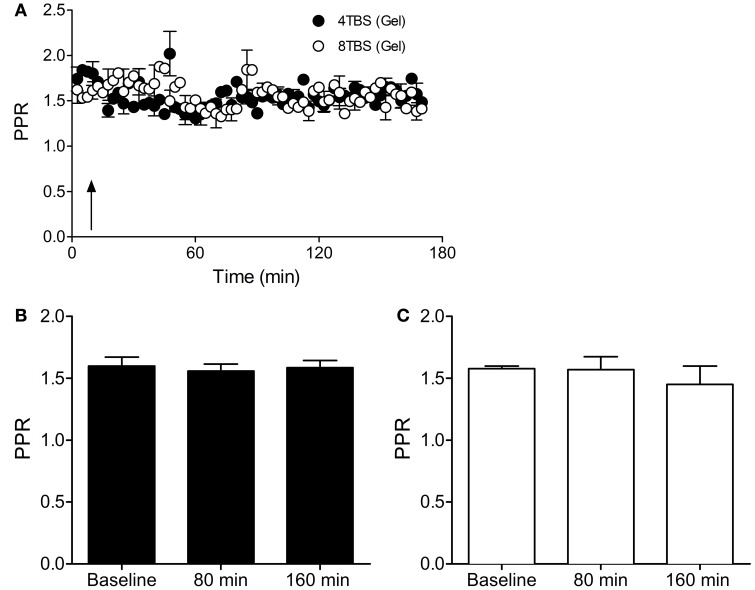
**Inhibition of postsynaptic translation with gelonin abolishes the enhanced Pr associated with LTP2 and LTP3. (A)** PPR data in slices stimulated with 4TBS and 8TBS (at time point indicated by arrow) with gelonin in the patch pipette internal solution. **(B)** Bar graph showing mean PPR during the first 10 min of recording (Baseline) and at 80 min and 160 min post-4TBS. PPR remained unchanged for the duration of the recording period. **(C)** Bar graph as for **(B)**, except for LTP3 data induced with 8TBS. PPR did not change over the entire recording period.

In further accordance with the FM 1-43 destaining data, inclusion of gelonin in the patch pipette internal solution abolished the elevated Pr that was associated with LTP2 (PPR Baseline = 1.60 ± 0.07, *n* = 9 cells; PPR 80 min = 1.56 ± 0.05, *n* = 9 cells; PPR 160 min = 1.58 ± 0.05, *n* = 9 cells; Figures 6A,B) and LTP3 (PPR Baseline = 1.57 ± 0.02, *n* = 5 cells; PPR 80 min = 1.57 ± 0.10, *n* = 5 cells; PPR 160 min = 1.45 ± 0.14, *n* = 5 cells; Figures 6A,C). These results support FM 1-43 destaining data suggesting that persistent forms of LTP recruit a presynaptic mode of expression that is dependent on translation in the postsynaptic compartment.

## Discussion

LTP1, 2, and 3 have previously been differentiated on the basis of persistence, Ca^2+^ signaling mechanisms, expression locus, electrophysiological requirements, and maintenance mechanisms (Abraham and Otani, 1991; Raymond et al., 2000; Raymond and Redman, 2002, 2006; Raymond, 2007, 2008; Johnstone and Raymond, 2011). The findings described here allow for further expansion of the LTP1, 2, and 3 model and provide additional evidence that certain classes of synapses are capable of supporting multiple discrete forms of LTP.

We now show that the enhanced exocytosis and P_r_ associated with LTP2 and LTP3 is eliminated in the presence of a translation inhibitor in the postsynaptic cell. Enhanced release was only abolished at terminals very closely apposed to the postsynaptic spines of treated neurons, whereas it remained intact at sites more distal to the treated dendrite. These data suggest the effects of gelonin were indeed confined to the treated cell and selectively affected the LTP response of terminals making synaptic contact with that cell.

### Synaptic cascade from postsynaptic activity to enhanced presynaptic release

Dendritic translation initiated by both 4TBS- and 8TBS-evoked Ca^2+^ signals is likely to result in the synthesis of many plasticity related proteins, one or more of which appears to be required for the presynaptic enhancement. Considering the dependence of presynaptic expression on NO signaling (Johnstone and Raymond, 2011), it is possible that translation of NO-related signaling proteins, such as NOS, could be important. NOS expression is known to be upregulated under certain stress conditions (De Oliveira et al., 2000; Yao et al., 2007), but has yet to be demonstrated during hippocampal synaptic plasticity. Furthermore, NOS mRNA has not been detected in CA1 dendrites and to date has only been observed in developing olfactory (Gibson and Nighorn, 2000) and optic (Koriyama et al., 2009) axons. Additionally, most reports suggest that NO exerts its influence only during *induction* of LTP (Schuman and Madison, 1991), therefore it seems likely that NO signaling will be upstream of the postsynaptic translation. Indeed, existing evidence suggests that NO is an important regulator of mRNA stability, acting to either stabilize or destabilize certain genes (Bouton and Demple, 2000; Ma et al., 2004). Under these conditions NO may play a critical role in regulating turnover of mRNA transcripts that are required to trigger changes in the kinetics of release.

These transcripts will likely encode proteins that are capable of signaling to the presynaptic cell, for example cell–cell adhesion molecules which are known to be crucial for some forms of LTP (Dityatev et al., 2008) and are important for regulating exocytosis (Saghatelyan et al., 2004; Dityatev et al., 2008). Alternatively dendritic translation of proteins that act as a second retrograde signal might be important. Given the existing data supporting a role for BDNF in presynaptic expression of TBS-induced LTP (Zakharenko et al., 2003), the preponderance of BDNF transcripts in hippocampal dendrites (Tongiorgi et al., 1997; An et al., 2008) and the observation that translation-dependent forms of LTP require BDNF (Bramham and Messaoudi, 2005) it is likely that postsynaptic translation of BDNF is a key process. Two recent studies provide further insight into the putative steps in the cascade. Henry et al. (2012) document the activity of the translation promoter mTOR complex 1 (mTORC1) in driving an activity-dependent, retrograde signaling cascade in hippocampal neurons that promotes enhanced neurotransmitter release from closely apposed presynaptic terminals. They further identify BDNF as key factor acting downstream of mTORC1 activation in establishing enhanced presynaptic function. Complementary to this, Penney and colleagues (2012) found that overexpression of TOR at the *Drosophila* neuromuscular junction is sufficient to trigger a strong retrograde increase in neurotransmitter release. These studies, in combination with the data we present here, suggest that postsynaptic activation of mTORC1 and subsequent retrograde action of BDNF is a likely pathway by which neurotransmitter release is enhanced during persistent forms of LTP.

### Experimental considerations

There are a small number of considerations that must be taken into account when interpreting the data presented here. Firstly, the results do not exclude a role for presynaptic translation in the expression of LTP2 and LTP3. It is possible that translation in the postsynaptic cell may trigger changes in the presynaptic terminal leading to further presynaptic translation of key proteins.

Secondly, it should be noted that gelonin has previously only been used as an inhibitor of synaptic translation in *Xenopus* (Zhang and Poo, 2002) and *Aplysia* (Sherff and Carew, 2004; Villareal et al., 2007) neuronal preparations, therefore, we cannot discount the possibility that it produces its inhibitory effects on LTP via different means in rodent hippocampus. It is also possible that gelonin may exert a local toxic effect which inhibits LTP induction in a manner that is independent of translation. Other non-toxic effects of gelonin might also be important. For example, gelonin may alter circuit excitability by interfering with sodium currents in the somatic compartment and/or reducing the amplitude or number of back-propagating action potentials during the induction stimulation. We consider these possibilities unlikely, however, given the qualitatively similar results we achieved with the membrane-permeable translation inhibitor anisomycin (Johnstone and Raymond, 2011) and the null effect of gelonin on LTP1 (Figure 1F) and normal synaptic transmission (Figure 1B).

Thirdly, the reduced magnitude of initial LTP2 and LTP3 that occurs with gelonin in the pipette (Figures 1B,C) merits a brief discussion. A possible explanation for this result is that gelonin might be acting upon products of so-called “immediate early genes.” For example, c-fos is known to be activated within approximately 5 min of stimulation (Flavell and Greenberg, 2008), and could conceivably have an effect on EPSC size. Other activity-regulated genes that directly regulate circuit connectivity may also play an important role in this early phase of gene expression. For example candidate plasticity gene 15 (Cpg15) is known to be regulated by activity and induces rapid AMPA receptor insertion, as well as initiating other changes that increase synaptic efficacy (Nedivi et al., 1998; Cantallops et al., 2000).

A final aspect of the experimental design that is important to consider is the rapid manner in which gelonin exerts its' inhibition on LTP. Because of the “wash-out” effect associated with whole-cell LTP recordings, induction with 4 or 8TBS occurred within 10–15 min of break in. It is unlikely that gelonin would diffuse to dendritic branches within this time period; therefore any early effect of gelonin on translation must be occurring within or near the somatic region. Additional effects of gelonin on any local, dendritic translation (and hence any changes in presynaptic release rate or probability associated with inhibition of dendritic translation) would only be apparent at later time points following LTP induction.

## Concluding remarks

Up until now it has been generally assumed that LTP-associated changes in postsynaptic protein expression underlie postsynaptic expression mechanisms, providing general support for postsynaptic LTP expression models. The data presented here demonstrate instead that alterations in protein synthesis within the postsynaptic compartment may also be critical in establishing the enhanced presynaptic release that is associated with some forms of LTP. This arrangement may allow for precise coordination of the degree and persistence of potentiation at a synapse, allowing only afferent input that induces sufficient postsynaptic Ca^2+^ to recruit changes in presynaptic release. It has been known for some time that protein synthesis is required for LTP to persist beyond a few hours. Our findings suggest that part of this requirement is related to the recruitment of a presynaptic expression mechanism that itself is strongly correlated with LTP persistence.

### Conflict of interest statement

The authors declare that the research was conducted in the absence of any commercial or financial relationships that could be construed as a potential conflict of interest.
